# Upright positioning facilitates the absorption of macular hole-related oedema

**DOI:** 10.1007/s00417-025-06757-1

**Published:** 2025-02-04

**Authors:** Vegard A. Forsaa, Birger Lindtjørn, Kristian Dahlø, Anastasia Ushakova, Jørgen Krohn

**Affiliations:** 1 Department of Ophthalmology, Stavanger University Hospital, PO box 8100, Stavanger, N-4068 Norway; 2 Department of Clinical Medicine, Section of Ophthalmology, University of Bergen, Bergen, Norway; 3 Department of Quality and Health Technology, University of Stavanger, Stavanger, Norway; 4 Department of Ophthalmology, Haukeland University Hospital, Bergen, Norway; 5 Department of Research, Section of Biostatistics, Stavanger University Hospital, Stavanger, Norway

**Keywords:** Circadian rhythm, Cystoid macular oedema, Diurnal rhythm, Macular oedema, Macular hole, Positioning

## Abstract

**Purpose:**

To investigate changes in macular hole-related oedema depending on positioning.

**Methods:**

Prospective interventional study of 40 patients with primary macular hole (MH). Optical coherence tomography scanning was done at 9 a.m., 1 p.m., and 3 p.m. Between the first and second scanning, the patients were instructed to stay upright, whereas they were positioned recumbent thereafter. Automated mean retinal thickness measurements were derived from the ETDRS grid for the central, parafoveal, and perifoveal subfields. Mean ocular perfusion pressure (MOPP) was calculated for all time points. Primary endpoints were changes in MH-related oedema from 9 a.m.−1 p.m., and from 1 p.m.−3 p.m.

**Results:**

In upright position from 9 a.m.−1 p.m., the mean parafoveal retinal thickness decreased from 362 μm (SD = 56) to 350 μm (SD = 51) (*P* < 0.001). The reduction of MH-related oedema when upright was positively correlated with a reduction in MOPP. Eyes with vitreomacular traction (VMT) exhibited significantly less reduction in MH-related oedema compared to eyes without VMT. In recumbent position from 1 p.m.−3 p.m., the mean parafoveal retinal thickness increased to 356 μm (SD = 52) (*P* = 0.002).

**Conclusion:**

MH-related oedema belongs to the non-vasogenic cystoid maculopathies. The decrease in MH-related oedema when upright and its positive correlation to a reduction in MOPP is therefore unexpected. In recumbent position, the situation is reversed, and the oedema increases. This may be related to subtle leakage from the retinal capillaries. The presence of VMT seems to counteract the resolution of the oedema. In a clinical setting, upright positioning after MH surgery facilitates absorption of the oedema which is beneficial for MH closure.

**Key messages:**

***What is known***:

- Macular hole formation is associated with cystoid macular oedema, possibly due to hydration of the outer retinal layers exposed to the hypotonic vitreous fluid. This oedema promotes the elevation of the hole edges from the retinal pigment epithelium.

***What is new***:

- Macular hole-related oedema decreases when the patients are upright and increases, in parallel with an increase in mean minimum macular hole diameter, when they are recumbent.
- The reduction of macular oedema is correlated with a reduction in mean ocular perfusion pressure, indicating that the oedema is influenced by subtle leakage from retinal capillaries.
- The results suggest that upright positioning might be beneficial in the early postoperative period of macular hole surgery.

## Introduction

Macular hole (MH) is a full thickness retinal defect in the fovea, with an annual incidence of 8 cases per 100 000 individuals [1]. MH most likely develop secondary to tractional forces exerted on the fovea by the posterior hyaloid [2]. The formation of a MH is associated with cystoid macular oedema (CMO), which is possibly caused by hydration of the outer retinal layers exposed to the hypotonic vitreous fluid [3, 4]. This fluid influx at the edges of the hole, combined with the rigidity of the internal limiting membrane, leads to an elevation of the hole edges from the retinal pigment epithelium (RPE). The interrupted contact between the neural retina and the RPE reduces the dehydrating effect of the RPE on the retina [5]. Hereby, a vicious circle is created, which effectively hinders the closure of the MH.

In CMO secondary to diabetes, retinal vein occlusions, and uveitis, the disturbed equilibrium of hydrostatic and osmotic pressures between the capillaries and the surrounding tissue can, as described by Starling’s law, lead to the accumulation of fluid in the retinal interstitium [6, 7]. In these conditions, there is often a breakdown of the inner blood retinal barrier (BRB). The leakage from altered retinal capillaries is affected by the intravascular hydrostatic pressure, i.e., the mean ocular perfusion pressure (MOPP). Substantial diurnal variations in the central retinal thickness (CRT) have been documented. In upright position during daytime, the CRT decreases, while an increase occurs at night when recumbent [8–11]. 

Position-related CRT changes are of particular interest in patients with MH, where the value of postoperative positioning regimens has long been a subject of debate. However, in contrast to the above-mentioned conditions, the current understanding of CMO in MH patients suggests no association with an inner BRB breakdown. This is reflected in the newly proposed term “non-vasogenic cystoid maculopathy” (NVCM), which defines maculopathies secondary to aetiologies other than vasogenic [12]. These conditions include, among others, MH, vitreomacular traction (VMT), epiretinal membrane (ERM), foveoschisis, and X-linked retinoschisis.

The objective of this study was to investigate the effects of different body positions on CMO associated with MH.

## Methods

### Study design and participants

This prospective interventional study was conducted at the Departments of Ophthalmology at Stavanger University Hospital and Haukeland University Hospital between February 2021 and April 2023. The inclusion criterion for the study was primary MH. The exclusion criteria included secondary MH, prior vitreoretinal surgery or any retinal vascular disease in the study eye, and age under 18 years.

The study was approved by the Regional Committee for Medical and Health Research Ethics, South-East B Norway (#154004), registered at ClinicalTrials.gov with the identifier NCTNCT04676217, and conducted according to the tenets of the Declaration of Helsinki. All patients provided written informed consent before participating in the study. The primary outcome measures were changes in automated retinal thickness measurements of the macular Early Treatment Diabetic Retinopathy Study (ETDRS) subfields [13]. 

### Study procedures and interventions

Eligible patients were examined at 9 a.m., 1 p.m., and 3 p.m. on the same day. Between 9 a.m. and 1 p.m., they were instructed to remain upright, either sitting, standing, or walking. From 1 p.m. to 3 p.m., they were positioned recumbent on the side of their MH eye. At each time point, the following examinations were conducted: intraocular pressure (IOP) in both eyes, measured with a handheld rebound tonometer (iCare IC200; Icare Finland Oy, Vantaa, Finland), average of three measurements of systolic and diastolic blood pressure, and macular optical coherence tomography (OCT) scanning. The two study sites used different OCT devices (Topcon DRI Triton; Topcon Corp, Tokyo, Japan, and Heidelberg Spectralis; Heidelberg Engineering, Heidelberg, Germany). Axial length was measured with an optical biometer (IOLMaster 700; Carl Zeiss Meditec AG, Jena, Germany).

The IOP and blood pressures were measured in sitting position at 9 a.m. and 1 p.m., and in recumbent position at 3 p.m. The OCT examination at 3 p.m. was performed with the patient sitting, with an approximate delay of 10–20 s from recumbent position. The MH eye was examined first, followed by the fellow eye.

The ETDRS grid of the OCT scan was manually centred on the MH. Automated retinal thickness and volume measurements were derived from the ETDRS grid and calculated for the central subfield, as well as the parafoveal (inner ring) and perifoveal (outer ring) subfields. The presence of VMT (defined as attachment of the posterior hyaloid to the area around the MH) and ERM was registered. The minimum and basal MH diameters were measured with the inbuilt calliper function. The minimum MH diameter was assessed at the narrowest hole point in the mid-retina, as a line roughly parallel to the RPE [14]. The measurements were performed independently by two of the authors and the mean diameter was recorded.

The MOPP in sitting and recumbent positions was derived from the mean brachial artery blood pressure (MAP) and IOP and calculated using the following equations [15].

$$\begin{array}{cc}Sitting:MOPP=2/3xMAP-IOP&Recumbent:MOPP=MAP-IOP\end{array}$$

### Statistical analysis and sample size estimation

Categorical data were summarised by numbers and proportions. Continuous normally distributed data were described by mean and standard deviation (SD) and by median and interquartile range (IQR) in cases of non-normality. In Tables 1 and 2, the mean values and SD are presented for all measurements for easier comparison. The Shapiro–Wilk test was used for assessing normal distribution. For comparison of continuous data, the Student’s t-test was employed for data with normal distribution, and for non-normally distributed data, the Mann–Whitney U test was used. For dependent variables, we used the paired sample t-test in case of normality and the Wilcoxon signed-rank test for non-normally distributed data. The correlation and corresponding confidence interval (CI) between changes in retinal thickness and the two variables MOPP and the time patients stood up in the morning were measured using the Pearson correlation coefficient. A two-sided *P* value ≤ 0.05 was considered statistically significant. Only statistically significant changes in OCT measurements exceeding the axial resolution of the OCT devices of 3.9 μm were considered relevant. The axial resolution of the systems is 3.9 μm for Heidelberg Spectralis, and 2.6 μm for Topcon DRI Triton. All statistical analyses were performed using the SPSS software, version 26 (IBM Corp., Armonk, NY, USA), and the graphs were generated with the R software, version 4.0.2 (R Foundation for Statistical Computing, Vienna, Austria). The sample size of 40 participants was determined by practical considerations. Power analysis for the two-sample Mann-Whitney U test indicates that a sample size of 40 participants with equal allocation is sufficient to detect a large effect size (Cohen’s d of 0.93) with 80% statistical power and a significance level of 5%.

**Table 1:** OCT measurements, blood pressure, intraocular pressure, and ocular perfusion pressure at different timepoints

	9 a.m.	1 *p*.m.	3 *p*.m.	∆ (9 a.m. – 1 *p*.m.)		∆ (1 *p*.m. – 3 *p*.m.)	
	Mean (SD)			Median difference (95% CI)	*P*	Median difference (95% CI)	*P*
Automated ETDRS measurements of MH eyes							
Central retinal thickness, µm	409 (69)	383 (60)	390 (64)	**−26 (−32; −20)**	**< 0.001**	**6 (2; 11)**	**0.008**
Parafoveal retinal thickness, µm	362 (56)	350 (51)	356 (52)	**−10 (−15; −5)**	< 0.001*	3 (1; 7)	0.002*
Perifoveal retinal thickness, µm	276 (26)	275 (24)	275 (27)	−0 (−2; 1)	0.977*	−1 (−2; 0)	0.163*
Total macular volume, mm3	8.41 (0.92)	8.31 (0.85)	8.33 (0.92)	−0.08 (−0.13; −0.05)	< 0.001*	0.02 (−0.01; 0.05)	0.252*
Automated ETDRS measurements of fellow eyes							
Central retinal thickness, µm	245 (29)	246 (29)	243 (29)	2 (0; 3)	0.017	−3 (−4; −2)	< 0.001
Parafoveal retinal thickness, µm	308 (26)	308 (25)	306 (25)	1 (−0; 1)	0.176	−2 (−2; −1)	< 0.001
Perifoveal retinal thickness, µm	269 (22)	268 (21)	267 (21)	−0 (−1; 1)	0.802	−1 (−1; −0)	0.010
Total macular volume, mm3	7.84 (0.64)	7.80 (0.60)	7.77 (0.61)	0.01 (−0.03; 0.03)	0.852	−0.03 (−0.05; −0.02)	0.002
Manual calliper-assisted measurements of MH eyes							
Minimum MH diameter, µm	568 (229)	564 (229)	574 (227)	−4 (−16; 8)	0.464	**9 (0; 18)**	**0.044**
Basal MH diameter, µm	1123 (418)	1108 (413)	1106 (405)	**−19 (−35; −2)**	**0.025**	−2 (−16; 11)	0.819
MAP, IOP, and calculated MOPP of MH and fellow eyes							
MAP, mm Hg	134 (14)	101 (9)	94 (12)	−32 (−35; −29)	0.001*	−6 (−11; −3)	< 0.001
MH eyes, IOP, mm Hg	14.6 (3.8)	15.0 (3.9)	19.1 (5.4)	0.5 (−0.0; 1.0)	0.231	4.0 (3.0; 5.0)	< 0.001*
MH eyes, MOPP, mm Hg	74.5 (10.6)	52.5 (7.3)	74.9 (12.2)	−21.6 (−23.8; −19.6)	< 0.001*	22.6 (18.7; 26.2)	< 0.001
Fellow eyes, IOP, mm Hg	14.4 (3.3)	14.8 (3.4)	17.9 (4.2)	0.5 (−0.5; 1.5)	0.312	3.5 (1.5; 5.0)	< 0.001
Fellow eyes, MOPP, mm Hg	74.7 (10.4)	52.5 (6.8)	76.2 (13.2)	−21.6 (−23.7; −19.8)	< 0.001*	24.0 (19.5; 28.0)	< 0.001*

Paired sample t-test for normally distributes values, otherwise Wilcoxon signed-rank test *

Statistically significant changes in OCT measurements exceeding the axial resolution of 3.9 μm used are indicated in bold. The axial resolution of the OCT systems was 3.9 μm for Heidelberg Spectralis, and 2.6 μm for Topcon DRI Triton

*CI* confidence interval, *ETDRS* Early Treatment Diabetic Retinopathy Study, *IOP* intraocular pressure, *MAP* mean brachial artery blood pressure, *MH* macular hole, *MOPP* mean ocular perfusion pressure, *OCT* optical coherence tomography, *SD* standard deviation

**Table 2:** Comparison of OCT measurements at different timepoints between macular hole eyes with and without vitreomacular traction and epiretinal membrane

	9 a.m. – 1 *p*.m. Mean (SD)		∆ (9 a.m. – 1 *p*.m.) Median difference (95% CI)	*P*	1 *p*.m. – 3 *p*.m. Mean (SD)		∆ (1 *p*.m. – 3 *p*.m.) Median difference (95% CI)	*P*
	**VMT**	**No VMT**			**VMT**	**No VMT**		
Change in central retinal thickness, µm	−15 (12)	−35 (15)	**−21 (−29; −11)**	**< 0.001**	6 (11)	7 (15)	0 (−7; 11)	0.957*
Change in parafoveal retinal thickness, µm	−7 (16)	−16 (13)	**−9 ( −18; −4)**	< 0.001*	4 (15)	7 (13)	3 (0; 10)	0.048*
Change in perifoveal retinal thickness, µm	0 (3)	−1 (5)	0 (−3; 2)	0.715*	−1 (2)	1 (6)	2 (0; 3)	0.059*
	**ERM**	**No ERM**			**ERM**	**No ERM**		
Change in central retinal thickness, µm	−29 (16)	−26 (18)	4 (−9; 17)	0.634	11 (10)	5 (14)	−9 (−16; 1)	0.079*
Change in parafoveal retinal thickness, µm	−10 (10)	−13 (16)	1 (−9; 5)	0.866*	4 (5)	7 (16)	−1 (−4; 4)	0.570*
Change in perifoveal retinal thickness, µm	0 (4)	−1 (4)	−1 (−4; 1)	0.286*	0 (6)	−1 (3)	0 (−2; 2)	0.747*

Student’s t-test for normally distributes values, otherwise Mann–Whitney U test*. Boldface indicates statistical significance

*CI* confidence interval, *ERM* epiretinal membrane, *SD* standard deviation, *VMT* vitreomacular traction

## Results

### Participants

Between April 2021 and May 2023, 40 patients were included, of whom 29 (73%) were female and 21 (53%) had MH in the right eye. The mean age at the time of examination was 67.3 years (SD, 6.5 years), and the median duration of symptoms was 12 weeks (IQR, 6–14 weeks). The mean minimum MH diameter was 568 μm (SD, 229 μm), and the mean basal diameter was 1123 μm (SD, 418 μm). Seventeen (43%) and 12 (30%) patients had VMT and ERM, respectively. The median axial length in the MH eyes and fellow eyes was 23.6 mm (IQR, 23.1–24.8 mm) and 23.5 mm (IQR, 23.2–24.8 mm), respectively. The mean time from when the patients stood up in the morning until the first OCT examination at 9 a.m. was 2 h and 30 min (SD, 41 min).

### Retinal thickness and volume

**Fig. 1:**
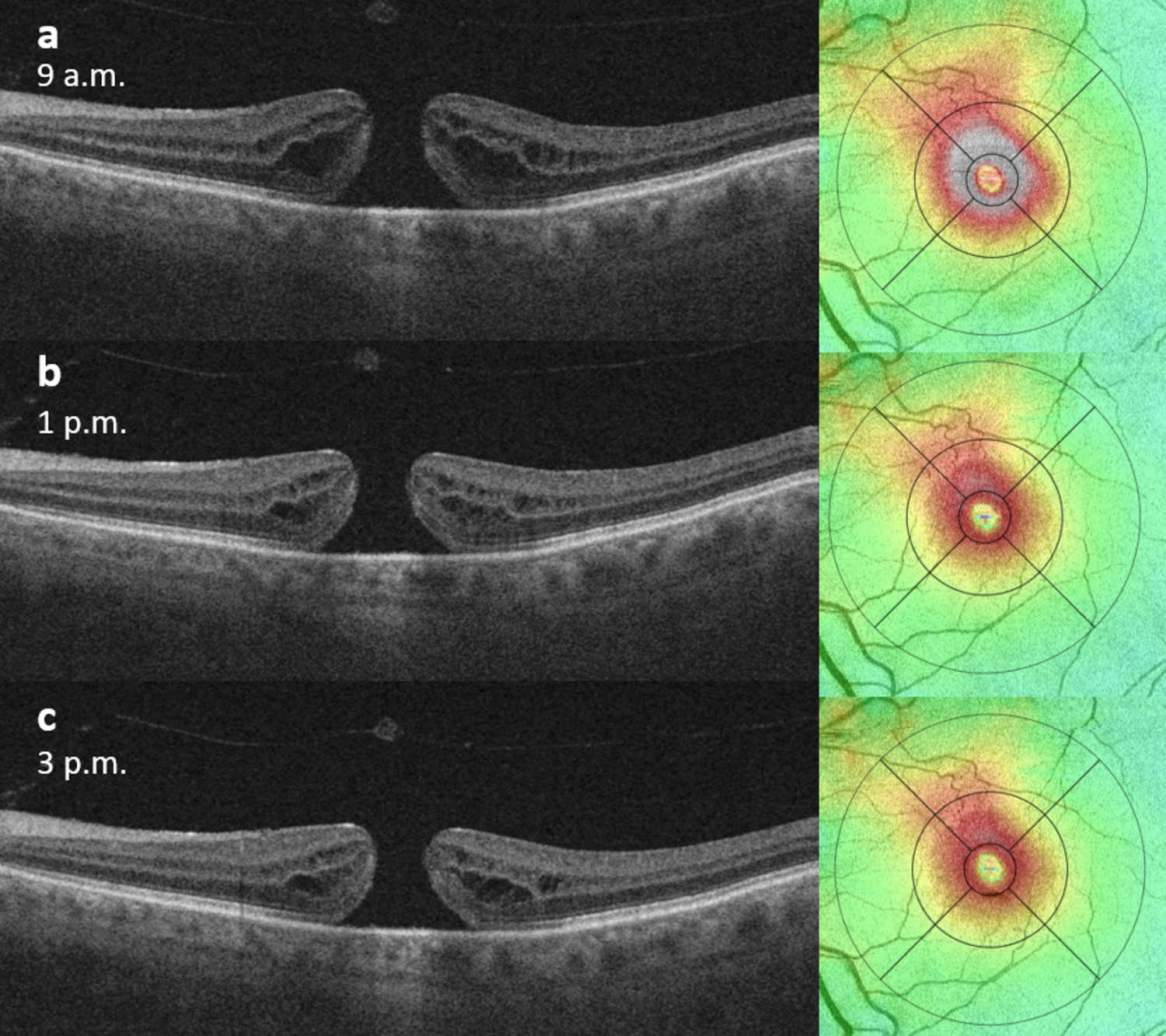
Optical coherence tomography B-scans and thickness maps of a study participant taken at different time points on the same day. The left section of the figures shows a horizontal cut through the centre of the macular hole, while the right section displays the corresponding ETDRS macular thickness map. **a** At 9 a.m., there is a significant oedema at the rim of the macular hole, as indicated by the grey colour in the central subfield and parafoveal subfields of the thickness map. **b** At 1 p.m., the macular oedema is less prominent. **c** At 3 p.m., the macular oedema remains largely unchanged

**Fig. 2:**
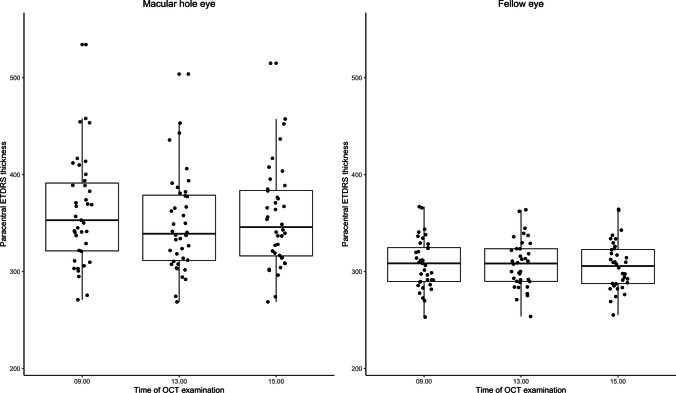
Box plots of paracentral ETDRS subfield thickness measured by optical coherence tomography (OCT) in the macular hole eyes and the fellow eyes when upright from 9 a.m. to 1 p.m., and after two hours in recumbent position at 3 p.m

The results of all automated OCT retinal thickness and volume measurements for both the MH eyes and the fellow eyes at each of the three timepoints are shown in Table 1. In the MH eyes, we observed a significant reduction in mean retinal thickness in the central subfield and the parafoveal ETDRS subfields from 9 a.m. to 1 p.m., when the participants were in upright position (Figs. 1 and 2).

The Pearson correlation coefficient revealed no significant correlation between this change in retinal thickness and the time patients stood up in the morning, neither for the central subfield (−0.12, *P* = 0.454; 95% CI, −0.004 to 0.002) nor for the parafoveal subfields (0.23, *P* = 0.182; 95% CI, −0.001 to 0.004). When recumbent from 1 p.m. to 3 p.m., the mean retinal thickness increased significantly in both the central subfield and the parafoveal subfields (Table 1; Fig. 2). No significant change occurred in the perifoveal ETDRS subfields. The mean ETDRS macular volume decreased significantly from 9 a.m. to 1 p.m. and remained unchanged thereafter.

From 9 a.m. to 1 p.m., a reduction in mean retinal thickness in the central subfield was found in 38 out of 40 cases, while two cases exhibited a small increase. From 1 p.m. to 3 p.m., a total of 23, 2, and 15 participants showed an increased, unchanged, or decreased mean parafoveal retinal thickness, respectively.

In recumbent position, from 1 p.m. to 3 p.m., the median change in retinal thickness in each of the four parafoveal ETDRS quadrants was as follows: superior 3 μm (IQR, −1–11), temporal 1 μm (IQR, −1–8), inferior 2 μm (IQR, −3–7), nasal 2 μm (IQR, −3–11).

In the fellow eyes, we observed a small, but significant increase in the mean retinal thickness of the central subfield from 9 a.m. to 1 p.m., and a significant decrease in the central and parafoveal subfields from 1 p.m. to 3 p.m. (Table 1; Fig. 2).

### Macular hole diameter

The minimum MH diameter remained unchanged when upright from 9 a.m. to 1 p.m., but increased significantly when recumbent from 1 p.m. to 3 p.m. The basal MH diameter decreased significantly from 9 a.m. to 1 p.m. and remained unchanged from 1 p.m. to 3 p.m. (Table 1).

### Vitreomacular traction and epiretinal membrane

**Fig. 3:**
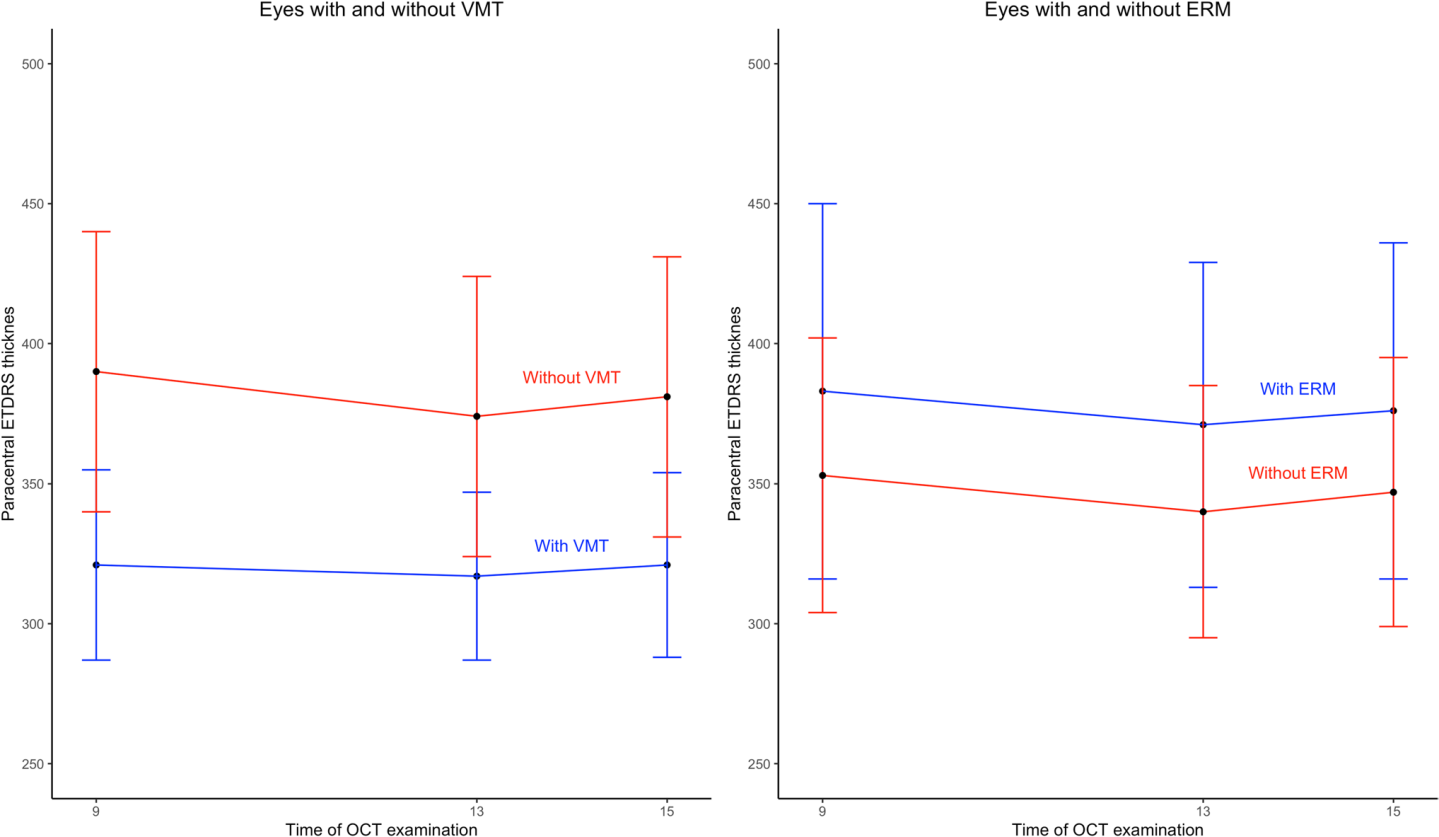
Graphs of paracentral ETDRS subfield thickness measured by optical coherence tomography (OCT) in eyes with and without vitreomacular traction (VMT) and epiretinal membrane (ERM) when upright from 9 a.m. to 1 p.m., and after two hours in recumbent position at 3 p.m

Comparisons of the mean changes in retinal thickness between MH eyes with and without VMT and ERM are presented in Table 2. In eyes with VMT, the mean retinal thickness in the central and parafoveal subfields decreased significantly less from 9 a.m. to 1 p.m. than in eyes without VMT (Fig. 3). The presence of ERM did not have a significant effect on changes in retinal thickness.

### Mean ocular perfusion pressure

The MOPP decreased significantly from 9 a.m. to 1 p.m. and increased back to the same level after two hours in recumbent position (Table 1). The Pearson correlation coefficient for changes in MOPP and mean retinal thickness in the central and parafoveal subfields from 9 a.m. to 1 p.m. was 0.21 (*P* = 0.209; 95% CI, −0.12 to 0.53) and 0.29 (*P* = 0.036; 95% CI, −0.28 to 0.60), respectively. For the changes in MOPP and mean retinal thickness in the central and parafoveal subfields in recumbent position from 1 p.m. to 3 p.m., the Pearson correlation coefficient was 0.23 (*P* = 0.075; 95% CI, **-**0.56 to 0.09) and 0.24 (*P* = 0.073; 95% CI, −0.55 to 0.08), respectively.

## Discussion

In this study, we have demonstrated a reduction in retinal thickness within the central and parafoveal ETDRS subfields from 9 a.m. to 1 p.m., when the participants were in upright position. When the participants were positioned recumbent from 1 p.m. to 3 p.m., an increased retinal thickness in the central and parafoveal subfields was observed. We also found a positive correlation between the reduction in MOPP and parafoveal retinal thickness in upright position from 9 a.m. to 1 p.m. This implies that macular oedema associated with MH behaves similarly to oedema related to diabetes mellitus, central retinal vein occlusion, and uveitis [8–11]. In these conditions, the BRB is damaged, resulting in leakage of fluid from the capillary bed. Such leakage will be affected by the MOPP, which is reported to decrease from early morning until after noon [16]. However, in conditions with NVCM like MH, where the capillaries are not known to leak, the role of MOPP in the observed alterations in macular thickness remains unknown. The intraretinal fluid in MH-related oedema probably originates from the vitreous and the retinal metabolism, independent of MOPP [17, 18]. On fluorescein angiography, leakage from retinal capillaries is normally not detected, but subtle leakage may evade detection due to active fluid absorption by the Müller cells and the RPE [5, 19]. In a previous study of 43 MH patients, a hyperfluorescent fluid cuff was seen in 47%, while there was absence of hyperfluorescence in 37% of the patients. A late fluorescein staining pattern, typical for CMO, was not found in any of the cases, but microvascular changes resembling microaneurysms or telangiectatic capillaries in the cuff area were noted in 12% of the patients [20]. In addition, a tractional trauma on the Müller cells could stimulate pro inflammatory factors and lead to gliosis and vascular leakage [12, 21, 22]. We propose that our finding of MOPP-related changes in MH-related oedema implies some degree of capillary leakage. The same pattern has also been observed in one other NVCM condition, namely X-linked retinoschisis [23, 24]. These studies also documented a daytime reduction in CRT.

The accumulation of fluid in a tissue depends not only on leakage or influx but also on the capacity of absorption [18, 25]. In MH cases, the absorption can be impaired in at least two ways. First, the retina surrounding the hole is detached, which means that the underlying RPE pump is overwhelmed by vitreous fluid. This induces a reduced capability of the RPE to clear fluid from the foveal tissue [26]. Second, the foveal Müller cells which dehydrate the inner retina by transporting water into the capillaries are traumatised. Impairment of the Müller cell ion pumps will lead to decreased fluid absorption, resulting in inner retinal cyst formation [27]. The increased hydrostatic pressure resulting from elevated MOPP may also impair the ability of the RPE and the Müller cells to transport fluid into the choroidal and retinal capillaries and lead to an increase in macular oedema [28, 29]. In the healthy fellow eyes, an opposite posture effect on retinal thickness could be observed. The thickness changes were minimal, but in line with earlier reports on circadian CRT patterns [30]. 

Compared with real-life MH surgery settings, our study has certain restrictions, as more detailed analyses involving various body positions, particularly face-down positioning, would be relevant. However, our primary focus was on comparing the major positions, upright versus recumbent, while also minimising the number of variables. Additionally, the potential impact of an intraocular gas bubble on our measurements remains unknown. Gravitational fluid shift has been proposed to have an impact on the CMO in diabetes and in MH eyes with gas tamponade [31, 32]. One would therefore expect a relatively higher increase in retinal thickness in the temporal ETDRS subfields because the participants were positioned recumbent on the side with their MH eye down. As there was no systematic variation in retinal thickness across the four ETDRS quadrants, we assume that gravitational forces have little or no effect on MH-related oedema under the studied conditions. However, since rapid displacements of oedema may occur, and the OCT was performed 10–20 s after transitioning from a recumbent to a sitting position, a gravitational impact on the MH-related oedema could not be completely ruled out.

Compared to the changes in retinal thickness, the minimum and basal MH diameters were less directly influenced by the body position. Nonetheless, the basal MH diameter decreased when upright whereas the minimum MH diameter increased when recumbent, indicating that upright positioning promotes MH closure.

It is debated whether the macular cystic cavities represent pseudocysts from tractional forces or oedematous cysts due to fluid influx. A combined picture, at least in cases with VMT is likely [26]. In established MH cases without VMT, fluctuations of cyst size have been observed, which is a behaviour typical for oedematous cysts. Vitreoretinal interface disorders, such as ERM and VMT, have the potential to influence how macular oedema responds to different body positions. We found that the presence of VMT significantly influenced this response. The reduction in macular oedema when upright was less prominent in eyes with VMT compared to those without VMT. It seems logical that anteroposterior traction exerted by the posterior hyaloid counteracts the reduction of macular oedema, but one would also expect that when recumbent, VMT should facilitate its aggravation. However, if the VMT also contributes to tangential traction and imparts some rigidity to the tissue, it may explain our findings. We found no effect of the presence or absence of ERM on changes in retinal thickness.

To the best of our knowledge, this is the first study assessing the impact of positioning on MH-related oedema. The low number of participants is a limitation of the study. In addition, the OCT scans at 3 p.m. were obtained from participants who transitioned to a sitting position seconds after being recumbent. This could potentially result in an undervaluation of the true increase in retinal thickness due to the rapid fluctuations in macular oedema [8]. Another possible bias is the shorter duration of recumbency compared to upright positioning, which could lead to an underestimation of the effect of recumbency. However, the assumed rapid changes in the amount of oedema makes this unlikely. During periods of upright positioning, the participants were allowed to sit, stand, or walk around. Consequently, their activity level was somewhat higher than during recumbency, which might have affected the results.

In summary, we have demonstrated that macular oedema related to MH decreases during the day when upright and increases, in parallel with an increase in mean minimum MH diameter, when recumbent. The reduction of macular oedema is correlated with a reduction in MOPP. This suggests that MH-related oedema is caused not only by influx from the vitreous and the retinal metabolism but also, to some extent, by vascular leakage and the ability of Müller cells and RPE to transport fluid back into the bloodstream. The results imply that maintaining a standing or sitting position, is beneficial in the early postoperative period of MH surgery. Further research is required to confirm these observations and to explore their implications for optimal positioning after MH surgery.

## Declarations

### Meeting presentation

This paper was presented in part at the XXXIV Meeting of the Club Jules Gonin, Palma de Mallorca, Spain, May 2024.

## References

[1] Forsaa VA, Lindtjorn B, Kvaloy JT, Froystein T, Krohn J (2017) Epidemiology and morphology of full-thickness macular holes. Acta Ophthalmol 96:397–404. 10.1111/aos.13618 29197164 10.1111/aos.13618

[2] Gass JD (1988) Idiopathic senile macular hole. Its early stages and pathogenesis. Arch Ophthalmol 106:629–639 3358729 10.1001/archopht.1988.01060130683026

[3] Woon WH, Greig D, Savage MD, Wilson MC, Grant CA, Mokete B, Bishop F (2015) Movement of the inner retina complex during the development of primary full-thickness macular holes: implications for hypotheses of pathogenesis. Graefe’s archive for clinical and experimental ophthalmology = Albrecht von Graefes Archiv fur klinische und experimentelle Ophthalmologie. 253:2103–2109. 10.1007/s00417-015-2951-0 10.1007/s00417-015-2951-0 25673252

[4] Tornambe PE (2003) Macular hole genesis: the hydration theory. Retina 23:421–424 12824853 10.1097/00006982-200306000-00028

[5] Govetto A, Sarraf D, Hubschman JP, Tadayoni R, Couturier A, Chehaibou I, Au A, Grondin C, Virgili G, Romano MR (2020) Distinctive mechanisms and patterns of Exudative Versus Tractional Intraretinal Cystoid spaces as seen with Multimodal Imaging. Am J Ophthalmol 212:43–56. 10.1016/j.ajo.2019.12.010 31862446 10.1016/j.ajo.2019.12.010

[6] Stefansson E (2009) Physiology of vitreous surgery. Graefe’s archive for clinical and experimental ophthalmology = Albrecht von Graefes Archiv fur klinische und experimentelle Ophthalmologie. 247:147–163. 10.1007/s00417-008-0980-7 10.1007/s00417-008-0980-7 19034481

[7] Haydinger CD, Ferreira LB, Williams KA, Smith JR (2023) Mechanisms of macular edema. Front Med (Lausanne) 10:1128811. 10.3389/fmed.2023.1128811 36960343 10.3389/fmed.2023.1128811 PMC10027768

[8] Munk MR, Kiss CG, Ekmekcioglu C, Huf W, Sulzbacher F, Gerendas B, Simader C, Schmidt-Erfurth U (2014) Influence of orthostasis and daytime on retinal thickness in uveitis-associated cystoid macular edema. Curr Eye Res 39:395–402. 10.3109/02713683.2013.845227 24215573 10.3109/02713683.2013.845227

[9] Larsen M, Wang M, Sander B (2005) Overnight thickness variation in diabetic macular edema. Investig Ophthalmol Vis Sci 46:2313–2316. 10.1167/iovs.04-0893 15980216 10.1167/iovs.04-0893

[10] Gupta B, Grewal J, Adewoyin T, Pelosini L, Williamson TH (2009) Diurnal variation of macular oedema in CRVO: prospective study. Graefe’s archive for clinical and experimental ophthalmology = Albrecht von Graefes Archiv fur klinische und experimentelle Ophthalmologie. 247:593–596. 10.1007/s00417-008-1011-4 10.1007/s00417-008-1011-4 19052771

[11] Frank RN, Schulz L, Abe K, Iezzi R (2004) Temporal variation in diabetic macular edema measured by optical coherence tomography. Ophthalmology 111:211–217. 10.1016/j.ophtha.2003.05.031 15019364 10.1016/j.ophtha.2003.05.031

[12] Gaudric A, Audo I, Vignal C, Couturier A, Boulanger-Scemama E, Tadayoni R, Cohen SY (2022) Non-vasogenic cystoid maculopathies. Prog Retin Eye Res 91:101092. 10.1016/j.preteyeres.2022.101092 35927124 10.1016/j.preteyeres.2022.101092

[13] (1991) Grading diabetic retinopathy from stereoscopic color fundus photographs–an extension of the modified Airlie House classification. ETDRS report number 10. Early Treatment Diabetic Retinopathy Study Research Group. Ophthalmology 98: 786–806 2062513

[14] Duker JS, Kaiser PK, Binder S, de Smet MD, Gaudric A, Reichel E, Sadda SR, Sebag J, Spaide RF, Stalmans P (2013) The International Vitreomacular Traction Study Group classification of vitreomacular adhesion, traction, and macular hole. Ophthalmology 120:2611–2619. 10.1016/j.ophtha.2013.07.042 24053995 10.1016/j.ophtha.2013.07.042

[15] Van Keer K, Breda JB, Pinto LA, Stalmans I, Vandewalle E (2016) Estimating Mean ocular perfusion pressure using Mean arterial pressure and intraocular pressure. Investig Ophthalmol Vis Sci 57:2260. 10.1167/iovs.16-19375 27124318 10.1167/iovs.16-19375

[16] Baek SU, Kim YK, Ha A, Kim YW, Lee J, Kim JS, Jeoung JW, Park KH (2019) Diurnal change of retinal vessel density and mean ocular perfusion pressure in patients with open-angle glaucoma. PLoS ONE 14:e0215684. 10.1371/journal.pone.0215684 31026291 10.1371/journal.pone.0215684 PMC6485647

[17] Bringmann A, Reichenbach A, Wiedemann P (2004) Pathomechanisms of cystoid macular edema. Ophthalmic Res 36:241–249. 10.1159/000081203 15583429 10.1159/000081203

[18] Cunha-Vaz JG, Travassos A (1984) Breakdown of the blood-retinal barriers and cystoid macular edema. Surv Ophthalmol 28 Suppl:485–492. 10.1016/0039-6257(84)90230-3 6379947 10.1016/0039-6257(84)90230-3

[19] Marmor MF (1999) Mechanisms of fluid accumulation in retinal edema. Doc Ophthalmol 97:239–249. 10.1023/a:1002192829817 10896337 10.1023/a:1002192829817

[20] Thompson JT, Hiner CJ, Glaser BM, Gordon AJ, Murphy RP, Sjaarda RN (1994) Fluorescein angiographic characteristics of macular holes before and after vitrectomy with transforming growth factor beta-2. Am J Ophthalmol 117:291–301. 10.1016/s0002-9394(14)73135-6 8129001 10.1016/s0002-9394(14)73135-6

[21] Schubert HD (1989) Cystoid macular edema: the apparent role of mechanical factors. Prog Clin Biol Res 312:277–291 2678144

[22] Lindqvist N, Liu Q, Zajadacz J, Franze K, Reichenbach A (2010) Retinal glial (Muller) cells: sensing and responding to tissue stretch. Investig Ophthalmol Vis Sci 51:1683–1690. 10.1167/iovs.09-4159 19892866 10.1167/iovs.09-4159

[23] Abalem MF, Musch DC, Birch DG, Pennesi ME, Heckenlively JR, Jayasundera T (2018) Diurnal variations of foveoschisis by optical coherence tomography in patients with RS1 X-linked juvenile retinoschisis. Ophthalmic Genet 39:437–442. 10.1080/13816810.2018.1466340 29902095 10.1080/13816810.2018.1466340 PMC6543537

[24] Rubinstein Y, Weiner C, Chetrit N, Newman H, Hecht I, Shoshany N, Pras E (2020) Effect of light and diurnal variation on macular thickness in X-linked retinoschisis: a case series. Graefe’s archive for clinical and experimental ophthalmology = Albrecht von Graefes Archiv fur klinische und experimentelle Ophthalmologie. 258:529–536. 10.1007/s00417-019-04578-7 10.1007/s00417-019-04578-7 31897705

[25] Lobo CL, Bernardes RC, Cunha-Vaz JG (2000) Alterations of the blood-retinal barrier and retinal thickness in preclinical retinopathy in subjects with type 2 diabetes. Arch Ophthalmol 118:1364–1369. 10.1001/archopht.118.10.1364 11030818 10.1001/archopht.118.10.1364

[26] Bringmann A, Unterlauft JD, Barth T, Wiedemann R, Rehak M, Wiedemann P (2021) Different modes of full-thickness macular hole formation. Exp Eye Res 202:108393. 10.1016/j.exer.2020.108393 33301774 10.1016/j.exer.2020.108393

[27] Reichenbach A, Wurm A, Pannicke T, Iandiev I, Wiedemann P, Bringmann A (2007) Muller cells as players in retinal degeneration and edema. Graefe’s archive for clinical and experimental ophthalmology = Albrecht von Graefes Archiv fur klinische und experimentelle Ophthalmologie. 245:627–636. 10.1007/s00417-006-0516-y 10.1007/s00417-006-0516-y 17219109

[28] Strauss O (2005) The retinal pigment epithelium in visual function. Physiol Rev 85:845–881. 10.1152/physrev.00021.2004 15987797 10.1152/physrev.00021.2004

[29] Negi A, Marmor MF (1986) Quantitative estimation of metabolic transport of subretinal fluid. Investig Ophthalmol Vis Sci 27:1564–1568 3771136

[30] Burfield HJ, Patel NB, Ostrin LA (2018) Ocular biometric diurnal rhythms in Emmetropic and myopic adults. Investig Ophthalmol Vis Sci 59:5176–5187. 10.1167/iovs.18-25389 30372744 10.1167/iovs.18-25389 PMC6203176

[31] Polito A, Polini G, Chiodini RG, Isola M, Soldano F, Bandello F (2007) Effect of posture on the diurnal variation in clinically significant diabetic macular edema. Investig Ophthalmol Vis Sci 48:3318–3323. 10.1167/iovs.06-1526 17591904 10.1167/iovs.06-1526

[32] Krohn J, Forsaa VA (2020) Is non-supine positioning superior to face-down positioning in macular hole surgery? Acta Ophthalmol 98:e558–e559. 10.1111/aos.14384 32096319 10.1111/aos.14384

